# On the Image Reconstruction of Capacitively Coupled Electrical Resistance Tomography (CCERT) with Entropy Priors

**DOI:** 10.3390/e25010148

**Published:** 2023-01-11

**Authors:** Zenglan Su, Manuchehr Soleimani, Yandan Jiang, Haifeng Ji, Baoliang Wang

**Affiliations:** 1 State Key Laboratory of Industrial Control Technology, College of Control Science and Engineering, Zhejiang University, Hangzhou 310027, China; 2 Engineering Tomography Laboratory (ETL), Department of Electronic and Electrical Engineering, University of Bath, Bath BA2 7AY, UK

**Keywords:** electrical tomography, capacitively coupled electrical resistance tomography (CCERT), image reconstruction, regularization, entropy

## Abstract

Regularization with priors is an effective approach to solve the ill-posed inverse problem of electrical tomography. Entropy priors have been proven to be promising in radiation tomography but have received less attention in the literature of electrical tomography. This work aims to investigate the image reconstruction of capacitively coupled electrical resistance tomography (CCERT) with entropy priors. Four types of entropy priors are introduced, including the image entropy, the projection entropy, the image-projection joint entropy, and the cross-entropy between the measurement projection and the forward projection. Correspondingly, objective functions with the four entropy priors are developed, where the first three are implemented under the maximum entropy strategy and the last one is implemented under the minimum cross-entropy strategy. Linear back-projection is adopted to obtain the initial image. The steepest descent method is utilized to optimize the objective function and obtain the final image. Experimental results show that the four entropy priors are effective in regularization of the ill-posed inverse problem of CCERT to obtain a reasonable solution. Compared with the initial image obtained by linear back projection, all the four entropy priors make sense in improving the image quality. Results also indicate that cross-entropy has the best performance among the four entropy priors in the image reconstruction of CCERT.

## 1. Introduction

The gas–liquid two-phase flow widely exists in industries such as the petroleum industry, chemical engineering, and refrigeration systems [1,2,3]. The parameter measurement of the gas–liquid two-phase flow is of critical significance for the system design, state monitoring, and safety control of industrial processes. 

Electrical resistance tomography (ERT) is a visualization technique to reconstruct 2D/3D cross-sectional conductivity distributions by a non-invasive electrode-sensing array [4,5,6]. Due to the advantages of low cost, high speed, and good safety, it has drawn much attention from researchers and has shown good application potential in the measurement of gas–liquid two-phase flow. However, the requirement of contact measurement leads to many adverse effects, such as the polarization, electrochemical corrosion, and contamination of the electrodes. To overcome these problems, capacitively coupled electrical resistance tomography (CCERT) was proposed as a contactless alternative of ERT [7]. It implements contactless measurement of the conductive medium by introducing the capacitive coupling principle, where a coupling capacitance is introduced to bridge the measurement path between the electrodes and the measured medium in the presence of the insulation layer between them. Research work has verified that CCERT can be applied to the field of multiphase flow [8,9]. However, as a relatively young technique, the research on CCERT is not mature enough, especially on image reconstruction algorithms. The imaging performance of CCERT still cannot satisfy the increasing requirement of practical application.

The image reconstruction of electrical tomography (ET) is a highly underdetermined and ill-posed inverse problem, as is CCERT [10,11,12]. The number of unknown variables (pixels) to be determined is much greater than the number of projections, which leads to a class of possible solutions. Meanwhile, the noise in measurement also causes randomness of the data. Previous researches have indicated that regularization is an effective way to alleviate these problems [13,14,15,16,17]. By incorporating the measurement data and a priori information, a reasonable solution consistent with both the data and the a priori knowledge can be obtained. The a priori information can be the deterministic support limit, band limit, positivity, or stochastic constraints, such as the probability density function [18,19]. However, the research on using regularization to improve the imaging performance of CCERT is very limited. It is necessary to seek valuable a priori information and develop effective regularization algorithms for CCERT.

Entropy priors have been proven to be promising in reconstructing positive images from limited and noisy data [20,21,22,23]. Generally, entropy priors are expected to satisfy the information theory, which can be used individually or combined with additional a priori information of the solution. There are several entropy estimators, such as information entropy, cross-entropy, conditional entropy, and joint entropy [24]. The entropy priors have been used with success in radiation tomography [25,26,27], such as X-ray tomography, γ-ray tomography [28,29], positron emission tomography [30,31], and optical diffraction tomography [32]. However, research on the application of entropy priors in ET are still limited [33,34,35], especially in CCERT. More research is needed to explore the potential of entropy priors in the image reconstruction of CCERT.

This work aims to investigate the regularization-based image reconstruction performance of CCERT with entropy priors. Under the maximum entropy (ME) strategy, three entropy priors, including the image entropy, the projection entropy, and the image-projection joint entropy, are introduced. Under the minimum cross-entropy (MCE) principle, the cross-entropy between the measured projection and the forward projection is introduced. The four entropy priors are used as the regularization terms to develop four corresponding objective functions. To solve the optimization problems expressed by the objective functions, linear back projection (LBP) is adopted to obtain the initial image and the steepest descent method is used to obtain the final image. Image reconstruction experiments will be carried out to verify the effectiveness of the four entropy priors in the image reconstruction of CCERT. The imaging performance of CCERT with the four entropy priors will be compared and discussed.

The rest of this article is organized as follows. Section 2 presents the measurement principle of CCERT, the image reconstruction with regularization, and the methods to reconstruct the image with entropy priors. The experimental results using practical collected data are provided in Section 3. Section 4 concludes this article. 

## 2. Methods

### 2.1. Measurement Principle of CCERT

**Figure 1:**
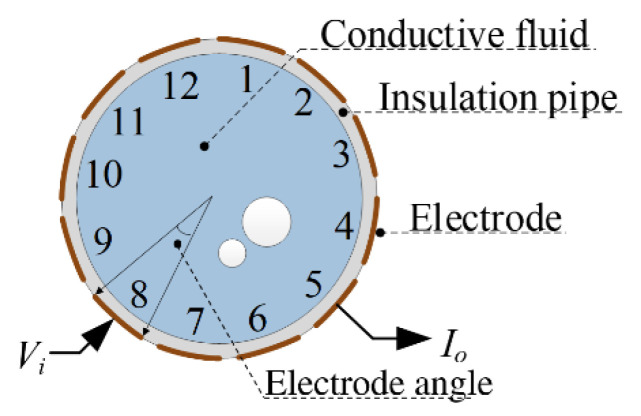
Construction of a 12-electrode CCERT sensor.

As mentioned, CCERT is a contactless technique. Figure 1 shows the construction of a 12-electrode CCERT sensor for the typical application of gas–liquid two-phase flow, which consists of 12 electrodes, the insulation pipe, and the conductive fluid in the pipe. As can be seen from Figure 1, the electrodes of CCERT are attached to the outer wall of the insulation pipe, not in direct contact with the fluid. 

**Figure 2:**
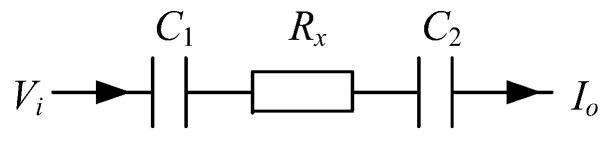
The simplified measurement path of an electrode pair.

According to the capacitive coupling principle, for each electrode, a capacitance will be formed by the electrode, the insulation pipe, and the conductive fluid. When a measurement electrode pair, including an excitation electrode and a detection electrode, is selected, a measurement path will be formed by two coupling capacitances and the equivalent resistance of the fluid between the two electrodes. Figure 2 shows the simplified measurement path of an electrode pair, where $C_{1}$ is formed by the excitation electrode, the insulation pipe, and the fluid; $C_{2}$ is formed by the detection electrode, the insulation pipe, and the fluid; $R_{x}$ is the equivalent resistance of the fluid. When an excitation voltage signal $V_{i}$ is applied to the excitation electrode, the equivalent resistance can be obtained by measuring the output current signal $I_{o}$ on the detection electrode. In a complete measurement cycle, a set of 66 equivalent resistances will be obtained for further image reconstruction. First, electrode 1 is selected as the excitation electrode and the equivalent resistances between electrode pairs 1–2, 1–3, …, 1–12 are obtained. Then, electrode 2 is selected as the excitation electrode and the equivalent resistances between electrode pairs 2–3, 2–4, …, 2–12 are obtained. This continues until electrode 11 is selected as the excitation electrode and the equivalent resistance between electrode pair 11–12 is obtained. During the measurement, the rest electrodes other than the measurement electrode pair are kept at the floating state. 

According to the Maxwell’s equations, the field of the sensor can be modeled as

(1) ∇·((σ(x,y)+jωε(x,y))∇φ(x,y))=0     (x,y)⊆Ω

where the sensing area is defined as Ω. σ(x,y), ε(x,y) and φ(x,y) are the conductivity, permittivity, and potential at the spatial point with coordinates (x,y) in Ω, respectively.

According to the typical measurement strategy of CCERT, two electrodes are selected as the excitation electrode and the detection electrode respectively, and the rest electrodes are at floating potential. The boundary conditions of Equation (1) are

(2) $$\left\{\begin{array}{c} \varphi_{a}\left(x,y\right)=V_{i}\;\;\;\;\;\;\;\;\;\;\;\;\left(x,y\right)\subseteq \Gamma_{a}\\ \varphi_{b}\left(x,y\right)=0\;\;\;\;\;\;\;\;\;\;\;\;\left(x,y\right)\subseteq \Gamma_{b}\\ \partial \varphi_{c}\left(x,y\right)/\partial \vec{n}=0\;\;\;\;\left(x,y\right)\subseteq \Gamma_{c},\left(c\neq a,b\right) \end{array}\right.$$

where $V_{i}$ is the amplitude of the excitation AC voltage source. a, b, and c represent the excitation electrode, the detection electrode, and the floating electrode, respectively. $\Gamma_{a}$, $\Gamma_{b}$, and $\Gamma_{c}$ represent the spatial regions of the excitation electrode, the detection electrode, and the floating electrodes. $\vec{n}$ denotes the outward unit normal vector.

### 2.2. Image Reconstruction with Regularization

CCERT includes two major computational problems, the forward problem and the inverse problem. The forward problem is to determine the inter-electrode resistances from the conductivity distribution in the sensing area satisfying Equations (1) and (2). The inverse problem is to determine the conductivity distribution from the resistance measurements in the form of a visual image, i.e., the image reconstruction. Therefore, the image reconstruction corresponds to solving the following inverse problem

(3) λ=Sg

where $\lambda ={\left[\lambda_{1}\;\lambda_{2}\ldots \;\lambda_{m}\ldots \;\lambda_{M}\right]}^{T}$ is the projection vector calculated from the resistance measurements. The number of resistance measurements is M=66, and the sensitivity matrix is $S={\left[s_{mn}\right]}_{M\times N}$. The number of elements/pixels in the sensing area is N. In this work, 32×32 square elements are used to mesh the sensing area. The image vector is $g={\left[g_{1}\;g_{2}\ldots \;g_{n}\ldots \;g_{N}\right]}^{T}$ which reflects the conductivity distribution in the sensing area. 

In detail, the mth projection $\lambda_{m}$ is calculated from the mth resistance measurement as

(4) $$\lambda_{m}=\frac{R_{m}-R_{m}^{0}}{R_{m}^{0}}$$

where $R_{m}^{0}$ is the mth resistance measurement obtained when the pipe is full of the liquid phase. $R_{m}$ is the mth resistance measurement obtained under the unknown distribution to be reconstructed. m=1, 2, …, M.

The sensitivity matrix is also known as the Jacobian matrix, which is the sensitivity of the resistance measurement to the conductivity change of the sensing area. Here, the sensitivity matrix is calculated by simulation based on the finite element method (FEM). The software ‘COMSOL Multiphysics’ and ‘MATLAB’ are used to implement the simulation. The sensitivity of the mth resistance measurement to the conductivity change of the nth element/pixel is defined as

(5) $$s_{mn}=\frac{R_{mn}-R_{m}^{0}}{R_{m}^{0}\Delta \sigma }$$

where $R_{m}^{0}$ is the mth resistance measurement when the pipe is full of the liquid phase ($\sigma =\sigma_{1},\;\varepsilon =\varepsilon_{1}$). $R_{mn}$ is the mth resistance measurement when the nth element changes from the liquid phase to the gas phase ($\sigma =\sigma_{2},\;\varepsilon =\varepsilon_{2}$). Δσ is the conductivity change, i.e., $\Delta \sigma =\sigma_{1}-\sigma_{2}$. m=1, 2, …, M and n=1, 2, …, N.

As mentioned, the number of unknown variables N is much larger than the number of resistance measurements M, which means that the inverse of S does not exist and Equation (3) cannot be solved analytically. Moreover, in practical cases, the resistance measurement data is mixed with noise. Equation (3) is then expressed as

(6) λ=Sg+e

where $e={\left[e_{1}\;e_{2}\ldots \;e_{m}\ldots \;e_{M}\right]}^{T}$ is the resistance measurement error vector.

A common method to solve Equation (3), i.e., Equation (6) in the real case, is to find the g that minimizes the residual between Sg and λ, which can be described as the following objective function

(7) $$\frac{1}{2}\|Sg-{\lambda \|}^{2}$$

Regularization provides an approach to solve the ill-posed problem and determine the solution from a constraint set of solutions using a priori information. By introducing regularization, the objective function becomes

(8) $$f\left(g\right)=\frac{1}{2}\|Sg-\lambda {\|}^{2}+\mu G\left(g\right)$$

where μ is the regularization parameter and G(g) is the regularization term. 

### 2.3. Image Reconstruction with Entropy Priors

Entropy is used to measure the uncertainty of information in Shannon’s theory, which has many properties that agree with the intuitive principles of what a measure of information should be. Generally, higher entropy means more information. Therefore, maximum entropy (ME) is a potential strategy to improve the image quality. Cross-entropy is a measure of the dissimilarity between two data sets. Smaller cross-entropy means higher similarity between two datasets. In this way, minimum cross-entropy (MCE) provides another approach to the search for a better image result. To study the application of entropy regularization in CCERT, the above ME and MCE strategies are introduced to develop the regularization term G(g) in Equation (8).

#### 2.3.1. Regularization with Maximum Entropy (ME) Strategy

According to Shannon’s theory, the entropy H of source τ is calculated by

(9) $$H\left(\tau \right)=-\sum_{\tau }p\left(\tau \right)log\;\left(p\left(\tau \right)\right)$$

where p(τ) is the probability mass function of τ.

To maximize entropy H(τ) one has to minimize −H(τ). Let G(g)=−H(τ), then the objective function to be minimized in Equation (8) can be rewritten as

(10) $$\;\;f\left(g\right)=\frac{1}{2}\|Sg-\lambda {\|}^{2}-\mu H\left(\tau \right)$$

which is defined as the ME objective function in this work. With different entropy estimators, the ME prior H(τ) to be measured is different. Correspondingly, different ME objective functions are determined. By minimizing the ME objective functions, the estimated solutions with ME priors can be obtained, respectively. Here, three entropy estimators are introduced as the ME priors, including the image entropy, the projection entropy, and the image-projection joint entropy.

(1) Maximum Image Entropy (MIE)

Image entropy is a good choice of a priori information for image reconstruction, which is a measure of the amount of information contained in an image. According to the maximum entropy principle, the larger the image entropy is, the more information in the image is predicted. The image entropy is defined as

(11) $$H\left(g\right)=-\sum_{n=1}^{N}g_{n}log\;g_{n}$$

Let H(τ)=H(g), then the ME objective function under image entropy is defined as

(12) $$f_{MIE}\left(g\right)=\frac{1}{2}\|Sg-\lambda {\|}^{2}+\mu \left(\sum_{n=1}^{N}g_{n}log\;g_{n}\right)$$

(2) Maximum Projection Entropy (MPE)

The quality of the reconstructed image also reflects on the quality of its forward projection. Thus, maximizing the projection entropy is also reasonable. Considering the forward process according to Equation (3), each predicted image g has a forward projection Sg. Then the projection entropy under one predicted image can be defined as a conditional entropy

(13) $$H\left(Sg|g\right)=-\sum_{m=1}^{M}{\left(Sg\right)}_{m}log{\left(Sg\right)}_{m}$$

Let H(τ)=H(Sg|g), then the ME objective function under projection entropy is defined as

(14) $$f_{MPE}\left(g\right)=\frac{1}{2}\|Sg-\lambda {\|}^{2}+\mu \sum_{m=1}^{M}{\left(Sg\right)}_{m}log{\left(Sg\right)}_{m}$$

(3) Maximum Joint Entropy (MJE)

The joint entropy H(I, J) of a pair of discrete random variables (i, j) with a joint distribution p(i,j) is defined as

(15) $$H\left(I,\;J\right)=\sum_{i}\sum_{j}p\left(i,j\right)log\left(p\left(i,j\right)\right)$$

Referring to the chain principle in entropy, then

(16)  H(I, J)=H(I)+H(J|I)

Thus, the joint entropy of the predicted image and its forward projection can be introduced as a combination of the image entropy and the projection entropy, which can be calculated by

(17)  H(g,Sg)=H(g)+H(Sg|g)

Let H(τ)=H(g,Sg), then the ME objective function under joint entropy is defined as

(18) $$f_{MJE}\left(g\right)=\frac{1}{2}\|Sg-\lambda {\|}^{2}+\mu \left(\sum_{n=1}^{N}g_{n}log\;g_{n}+\sum_{m=1}^{M}{\left(Sg\right)}_{m}log{\left(Sg\right)}_{m}\right)$$

#### 2.3.2. Regularization with Minimum Cross-Entropy (MCE) Strategy

Cross-entropy evaluates the dissimilarity between two data sets. Larger cross-entropy means a greater degree of dissimilarity between the two data sets. In radiation tomography for biomedical applications, the minimization of the cross-entropy between the current estimate of the reconstructed image and the anatomical a priori images are used to guide the image reconstruction. In this work, for gas–liquid two-phase flow, the dissimilarity between the practical projection set from the resistance measurements λ and the forward projection from the current estimated image Sg is introduced as the entropy prior. The cross-entropy between λ and Sg can be described as

(19) $$L\left(g\right)=\sum_{m=1}^{M}\left[\lambda_{m}log\;\frac{\lambda_{m}}{{\left(Sg\right)}_{m}}-\lambda_{m}+{\left(Sg\right)}_{m}\right]$$

Let G(g)=L(g), the objective function under minimum cross-entropy is then

(20) $$f_{MCE}\left(g\right)=\frac{1}{2}\|Sg-\lambda {\|}^{2}+\mu \{\sum_{m=1}^{M}\left[\lambda_{m}log\;\frac{\lambda_{m}}{{\left(Sg\right)}_{m}}-\lambda_{m}+{\left(Sg\right)}_{m}\right]\}$$

Similarly, by minimizing the above equation, the estimated solution with minimum cross-entropy constraint can be obtained.

#### 2.3.3. Image Reconstruction Process

With the four objective functions in Equations (12), (14), (18) and (20), an estimated image $\hat{g}$ can be obtained by introducing optimization algorithms to minimize each objective function f(g). The estimated image can be described as

(21) $$\hat{g}=\arg\underset{g}{\min}f\left(g\right)$$

Optimization problems widely exist in engineering technologies, and finding the optimal solution is always the goal for researchers and engineers. Research works have been undertaken to seek effective optimization methods, and great achievements have been obtained. Currently, there are many useful methods to solve the nonlinear optimization problem [36,37], such as the steepest descent algorithm, the Newton’s algorithm, the conjugate gradient method, the intelligent optimization algorithms, etc. Among them, the steepest descent method has the best convergence characteristics and minimal single step computation. The conjugate gradient method and the Newton’s algorithm are applicable to the quadratic form optimization problem, such as the objective function in Equation (7). For nonquadratic problem, the conjugate gradient method cannot keep the searching direction conjugate as the number of iterations increase, and the Newton’s algorithm might not guarantee the value of the objective function to decline steadily. Therefore, the steepest descent method is adopted in this work to solve the optimization problem in Equation (21). The steps of the steepest descent method are concluded as follows:

1. Let the number of iterations k:=0 and initialize the image vector $g^{0}$.
2. Calculate the gradient $\nabla f\left(g^{0}\right)$ and set the initial iteration direction $d^{0}=-\nabla f\left(g^{0}\right)$.
3. Determine the step length by linear searching as $\alpha_{k}=\arg\underset{\alpha \geq 0}{\min}f\left(g^{k}+\alpha d^{k}\right)$.
4. Calculate the new image vector $g^{k+1}=g^{k}+\alpha^{k}d^{k}$.
5. Calculate the new gradient $\nabla f\left(g^{k+1}\right)$.
6. Update the iteration direction as $d^{k+1}=-\nabla f\left(g^{k+1}\right)$.
7. Set k:=k+1, if either of the termination conditions is satisfied, stop the iteration and let the final image vector to be $\hat{g}=g^{k+1}$. Otherwise, return to step 3.

Here, the termination conditions are:

(1) $\nabla f\left(g^{k}\right)=0$
(2) $\left|f\left(g^{k}\right)-f\left(g^{k+1}\right)\right|<\xi_{1}$
(3) k:=k+1> $\xi_{2}$

where the termination coefficients are set as $\xi_{1}=1\times 10^{-8}$ and $\xi_{2}=1000$.

The initial value of the solution is important in optimization. Here, the initial image $g^{0}$ is obtained by LBP, which is widely used as the first stage algorithm due to its simplicity and low computation cost. It reconstructs the image by back-projecting the boundary projections to the sensitivity matrix, which can be expressed as

(22) $$g_{n}^{0}=\frac{\sum_{m=1}^{M}\lambda_{m}s_{mn}}{\sum_{m=1}^{M}s_{mn}}$$

With the initial image obtained by LBP, the final image is reconstructed by the iterations of the steepest descent method.

## 3. Results and Discussion

### 3.1. Experimental Setup

**Figure 3:**
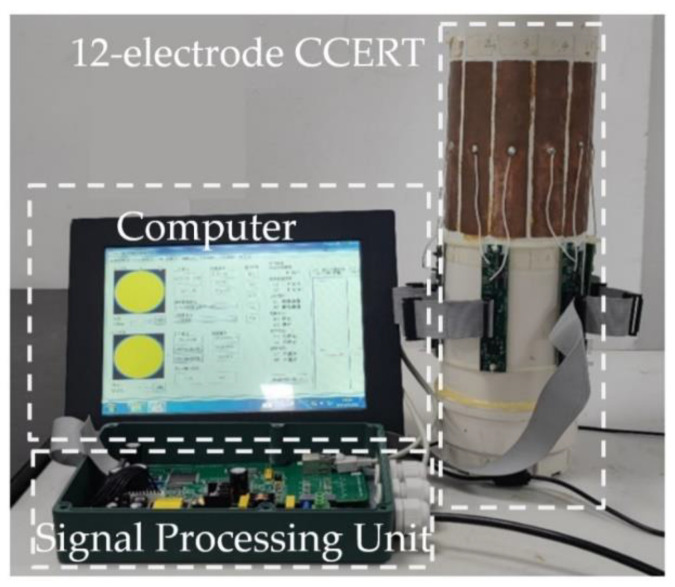
Photo of the experimental setup.

Figure 3 shows the experimental setup in this work, which is composed of a 12-electrode CCERT sensor, a signal processing unit, and a computer. The 12-electrode CCERT sensor is made up with a PVC plastic pipe and an electrode array. The height of the pipe is 400 cm and the outer diameter is 110 mm with the pipe wall thickness of 2 mm. The length of the electrodes is 125 mm, and the electrode angle in Figure 1 is 25 degrees (the width of the electrode is about 23 mm). The signal processing unit is for data acquisition, including the control of the excitation and measurement process, the calculation of the resistance measurements, and the communication with the computer. The computer is used to reconstruct the images with the resistance measurements.

To evaluate the quality of the reconstructed image, relative image error (RIE) and image correlation coefficient (ICC) are introduced, which are commonly used in the literature of ET [38,39,40]. The smaller RIE and larger ICC mean better image quality. They are calculated by:

(23) $$\;\;RIE=\sqrt{\frac{\sum_{n=1}^{N}{\left(\hat{g_{n}}-g_{n}^{*}\right)}^{2}}{\sum_{n=1}^{N}{\left(g_{n}^{*}\right)}^{2}}}$$

(24) $$\;ICC=\frac{\sum_{n=1}^{N}\left(\hat{g_{n}}-\hat{g_{a}}\right)\left(g_{n}^{*}-g_{a}^{*}\right)}{\sqrt{\sum_{n=1}^{N}{\left(\hat{g_{n}}-\hat{g_{a}}\right)}^{2}{\left(g_{n}^{*}-g_{a}^{*}\right)}^{2}}}$$

where $\hat{g_{n}}$ is the gray value of the nth pixel in the reconstructed image $\hat{g}$, $g_{n}^{*}$ is the gray value of the nth pixel in the ground truth $g^{*}\;$(will be introduced in Section 3.2), $\hat{g_{a}}$ and $g_{a}^{*}$ denote the average value of the gray value of them, respectively.

Tikhonov regularization algorithm is a typical representative in the regularization-based reconstruction family. Therefore, it is used for comparison in this work. Tikhonov regularization includes a *l*_2_-norm smooth constraint, where the typical form of G(g) is $G\left(g\right)=\|g{\|}^{2}$. With the standard Tikhonov procedure, the solution of Equation (8) is

(25) $$\hat{g}={\left(S^{T}S+\mu E\right)}^{-1}S^{T}\lambda$$

where E is an identity matrix. $\hat{g}$ is the estimated solution constrained by the Tikhonov regularization. Tikhonov is a one-step method that introduces a trade-off between fitting the data exactly to determine a solution and limiting the value of the solution. The trade-off is controlled by the regularization parameter μ, which is with positive value. 

### 3.2. Experimental Results and Discussion

**Figure 4:**
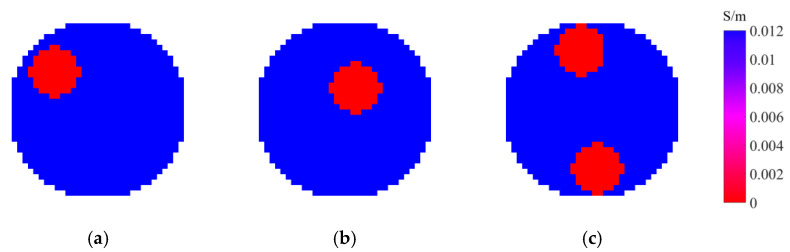
Ground truth of the tested scenarios. (**a**) Scenario 1 (S1). (**b**) Scenario 2 (S2). (**c**) Scenario 3 (S3).

**Figure 5:**
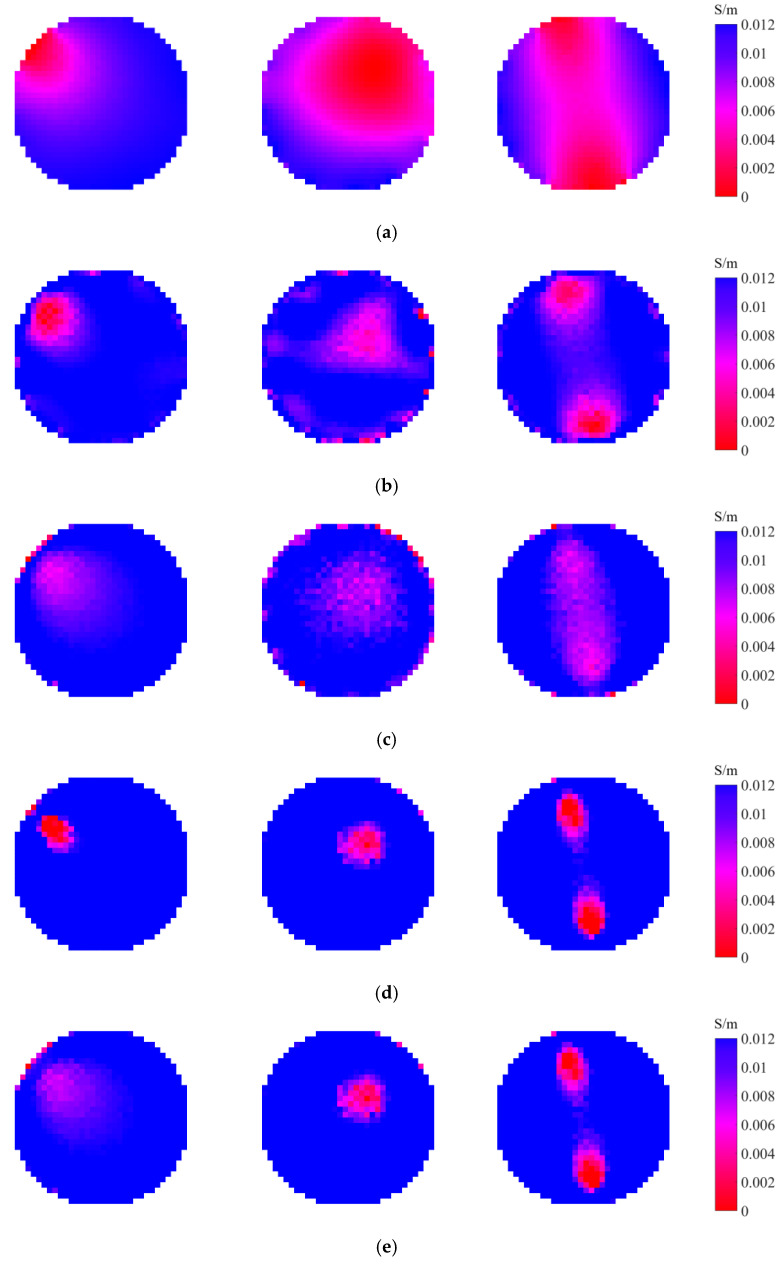

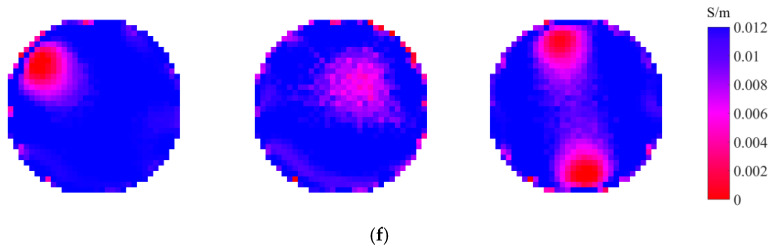
Image reconstruction results obtained by different algorithms. (**a**) LBP. (**b**) Tikhonov. (**c**) MIE. (**d**) MPE. (**e**) MJE. (**f**) MCE.

**Figure 6:**
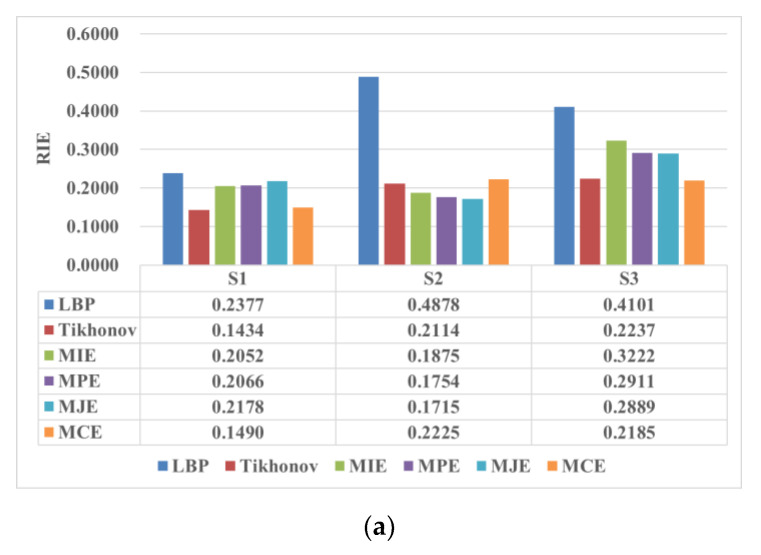

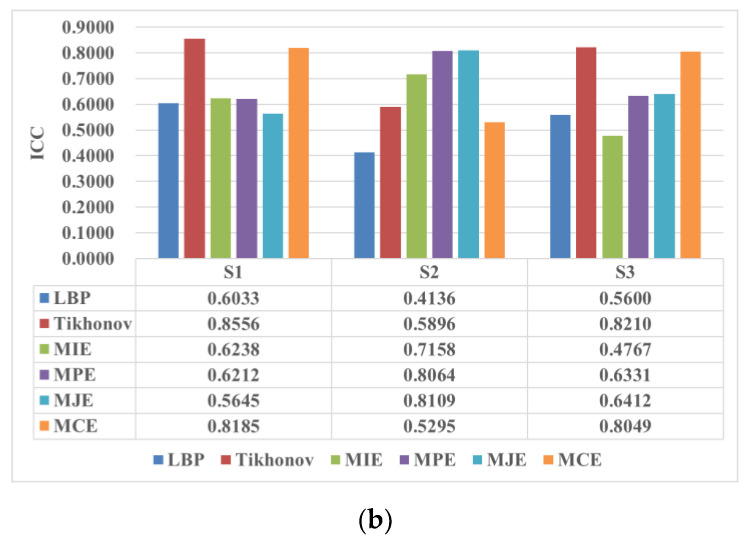
The image quality indexes. (**a**) RIE (**b**) ICC.

Figure 4 shows the ground truth of the tested scenarios, which is the practical distributions to be reconstructed. There are three scenarios S1 to S3 to be evaluated, as shown in Figure 4, where tap water (conductivity $\sigma_{1}=0.012\;S/m$ and relative permittivity $\varepsilon_{1}=78$) and plastic rod(s) (conductivity $\sigma_{2}=0\;S/m$ and relative permittivity $\varepsilon_{2}=3$) with the diameter of 30 mm are used to simulate the background and the target(s), respectively. Scenarios S1 and S2 are for one target at different positions and scenario S3 is for two targets. Figure 5 presents the experimental results obtained by the investigated four entropy priors, including the maximum image entropy (MIE), maximum projection entropy (MPE), maximum joint entropy (MJE), and minimum cross-entropy (MCE), which are listed in a sequence. As mentioned, LBP is utilized to get the initial image, while Tikhonov is a typical conventional algorithm using the $l_{2}$-norm regularization. Thus, the results obtained by LBP and Tikhonov are also listed in Figure 5 for comparison to evaluate the performance of the four-entropy regularization. Figure 6 shows the corresponding image quality indexes of the reconstructed images. There are 856 square elements in the region of interest (the gas–liquid two-phase flow in the circular pipe), so only 856 pixels are displayed in the images.

As can be seen in Figure 5, the initial images obtained by LBP have a large artifact in all three test cases, which blurs the positions and sizes of the targets. However, the artifact in the images reconstructed by MIE, MPE, MJE, and MCE have been mostly eliminated. The four entropy priors are capable to obtain a more reasonable and accurate prediction of the targets than LBP, which is also illustrated quantitively in Figure 6. All the images reconstructed by the four entropy priors are in accord with the ground truth. This verifies the effectiveness of the entropy priors and indicates their potential in the image reconstruction of CCERT.

Among the three ME priors, the two priors derived from projection entropy (MPE and MJE) are good at obviating the artifact and keeping relative clear boundaries of the targets. Figure 6 shows that MPE and MJE perform better than the other priors in S2, including the Tikhonov regularization. However, MPE and MJE struggle with recovering the size of the targets. It is observed that the targets in the images reconstructed by MPE and MJE are somewhat shrunken in size, thus they have poorer quantitative performance in S1 and S3, as shown in Figure 6. For MIE, the boundaries of the images are blurry, probably because the artifact means more information to some extent, resulting in larger image entropy. The comparison of the results of MPE and MJE also indicates that using the image entropy as the regularization term has a smaller impact on the objective function. The images reconstructed by the MJE and the MPE are comparable, which indicates that the contribution of MPE is dominating and the contribution of MIE is much smaller. To sum up, for ME priors, MIE contributes less in the objective function, while MPE and MJE eliminate the artifact well but out of balance in the target size.

Compared with the three ME priors, the MCE prior shows a better trade-off between removing the artifact and recovering the size of targets. The MCE prior performs better than the ME priors in the reconstruction of S1 and S3, as shown in Figure 6. The reason might be that the ME priors have no reference to the real measurements, so they are only responsible for the information. However, the cross-entropy provides a measure of dissimilarity between the measured projection and the forward projection, which bridges the information and the measurements. It is reasonable that a closer forward projection to the measurement projection means closer reconstructed image to the ground truth. However, compared with the classical Tikhonov regularization, the performance of MCE prior has no advantage. The images reconstructed by the MCE prior are comparable to those reconstructed by Tikhonov regularization.

## 4. Conclusions

This work investigates the image reconstruction of CCERT by introducing entropy priors as the regularization terms. Four types of entropy priors (MIE, MPE, MJE, and MCE) are introduced. By developing the objective function for each entropy prior, the combination of LBP and the steepest descent method is adopted to optimize the objective function. Experiments are carried out and the performances of the four entropy priors are compared.

Results show that all the images reconstructed by the four entropy priors are in accord with the ground truth. Compared with the initial image obtained by LBP, regularization with the entropy priors is an effective way to improve the image quality. Among the four entropy priors, the MCE prior has the overall best performance in the trade-off between eliminating the artifact and recovering the size of the targets, while the MIE prior has the poorest performance. MPE and MJE behave the best in eliminating artifacts, but struggle with keeping the size of the reconstructed targets. It is found that these two priors have good performance in reconstructing the target close to the low-sensitivity center of the sensing area.

New knowledge and experience have been obtained in this investigation which can provide useful references for further research work. Although the research results show the potential of entropy priors in the image reconstruction of CCERT, the imaging performance of the entropy priors have no advantage over that of the classical Tikhonov regularization. There is still much space for progress in future research on seeking more effective a priori information to take advantage of entropy. For industrial multi-phase flow, if other prior information can be incorporated into the entropy prior, such as dynamic modelling data obtained by simulation or good-quality multi-frequency images obtained by the CCERT system, the informatic nature of entropy is thought to make more sense.

## Data Availability

Not available due to privacy.
